# Envelope-Specific Recognition Patterns of HIV Vaccine-Induced IgG Antibodies Are Linked to Immunogen Structure and Sequence

**DOI:** 10.3389/fimmu.2019.00717

**Published:** 2019-04-24

**Authors:** Yuka Nadai, Kathrin Held, Sarah Joseph, Mohamed I. M. Ahmed, Verena S. Hoffmann, David Peterhoff, Marco Missanga, Asli Bauer, Agricola Joachim, Ulf Reimer, Johannes Zerweck, Sheena McCormack, Alethea V. Cope, Roger Tatoud, Robin J. Shattock, Merlin Lee Robb, Eric G. Sandstroem, Michael Hoelscher, Leonard Maboko, Muhammad Bakari, Arne Kroidl, Ralf Wagner, Jonathan Weber, Georgios Pollakis, Christof Geldmacher

**Affiliations:** ^1^Division of Infectious Diseases and Tropical Medicine, University Hospital, LMU Munich, Munich, Germany; ^2^German Center for Infection Research (DZIF), Partner Site Munich, Munich, Germany; ^3^MRC Clinical Trials Unit at UCL, London, United Kingdom; ^4^Institute of Medical Microbiology and Hygiene, University Regensburg, Regensburg, Germany; ^5^NIMR-Mbeya Medical Research Center, Mbeya, Tanzania; ^6^Muhimbili University of Health and Allied Sciences, Dar es Salaam, Tanzania; ^7^JPT Peptide Technologies, Berlin, Germany; ^8^Department of Medicine, Imperial College London, London, United Kingdom; ^9^US Military HIV Research Program, Silver Spring, MD, United States; ^10^Department of Clinical Science and Education, Karolinska Institutet at Södersjukhuset, Stockholm, Sweden; ^11^Institute of Clinical Microbiology and Hygiene, University Hospital, Regensburg, Germany; ^12^Institute of Global Health (CIMI), University of Liverpool, Liverpool, United Kingdom

**Keywords:** HIV, vaccine, envelope-specific antibodies, epitope variant recognition, immunogen structure, immunogen sequence

## Abstract

**Background:** A better understanding of the parameters influencing vaccine-induced IgG recognition of individual antigenic regions and their variants within the HIV Envelope protein (Env) can help to improve design of preventive HIV vaccines.

**Methods:** Env-specific IgG responses were mapped in samples of the UKHVC003 Standard Group (UK003SG, *n* = 11 from UK) and TaMoVac01 (TMV01, *n* = 17 from Tanzania) HIV vaccine trials. Both trials consisted of three immunizations with DNA, followed by two boosts with recombinant Modified Vaccinia Virus Ankara (MVA), either mediating secretion of gp120 (UK003SG) or the presentation of cell membrane bound gp150 envelopes (TMV01) from infected cells, and an additional two boosts with 5 μg of CN54gp140 protein adjuvanted with glucopyranosyl lipid adjuvant (GLA). Env immunogen sequences in UK003SG were solely based on the clade C isolate CN54, whereas in TMV01 these were based on clades A, C, B, and CRF01AE. The peptide microarray included 8 globally representative Env sequences, CN54gp140 and the MVA-encoded Env immunogens from both trials, as well as additional peptide variants for hot spots of immune recognition.

**Results:** After the second MVA boost, UK003SG vaccinees almost exclusively targeted linear, non-glycosylated antigenic regions located in the inter-gp120 interface. In contrast, TMV01 recipients most strongly targeted the V2 region and an immunodominant region in gp41. The V3 region was frequently targeted in both trials, with a higher recognition magnitude for diverse antigenic variants observed in the UK003SG (*p* < 0.0001). After boosting with CN54gp140/GLA, the overall response magnitude increased with a more comparable recognition pattern of antigenic regions and variants between the two trials. Recognition of most immunodominant regions within gp120 remained significantly stronger in UK003SG, whereas V2-region recognition was not boosted in either group.

**Conclusions:** IgG recognition of linear antigenic Env regions differed between the two trials particularly after the second MVA boost. Structural features of the MVA-encoded immunogens, such as secreted, monomeric gp120 vs. membrane-anchored, functional gp150, and differences in prime-boost immunogen sequence variability most probably contributed to these differences. Prime-boosting with multivalent Env immunogens during TMV01 did not improve variant cross-recognition of immunodominant peptide variants in the V3 region.

## Introduction

Development of an efficacious vaccine against the Human Immunodeficiency Virus-1 (HIV) is complicated by high variability of the HIV envelope glycoprotein (Env), and by the difficulty to induce broadly cross-reactive neutralizing HIV Env-specific antibody responses (1, 2). Vaccine-induced protection from HIV acquisition may nonetheless be possible in the absence of a strong HIV neutralizing antibody response; during the Rv144 trial, high IgG levels targeting the Env hypervariable regions 1 and 2 (V1V2) correlated with protection from HIV acquisition (3). Subsequent studies further mapped this V1V2-specific IgG response to a linear 15 mer peptide in the V2 region located in close proximity to the α4β7 integrin-binding motif (4, 5). IgG responses against linear peptides covering the highly immunogenic V3 region were also inversely correlated with infection risk in Rv144 in a subgroup analysis (4), whereas recognition of other, more conserved Env regions within gp120, showed no correlation with infection risk in Rv144 (4). While the protective mechanism of these V2- and V3- specific antibody responses is incompletely understood, the “sieve effect” observed in Rv144 break through infections provides solid evidence for the protective effect of immune responses targeting these hypervariable regions: Break through HIV strains in Rv144 vaccine recipients had specific point mutations in the targeted V2 and V3 epitopes compared to those in placebo recipients (6, 7). High levels of vaccine-induced Env-binding antibodies—and in particular those targeting the V2 region—also correlated with protection from AIDS virus acquisition during a well-controlled non-human primate study (8). Together these results emphasize that non-neutralizing antibody responses targeting vulnerable Env regions may mediate vaccine-induced immunity.

Properties inherent to different immunogens and vaccination strategies are likely to determine immune recognition of a specific region and its antigenic variants. A better understanding of the vaccine parameters influencing IgG recognition of different Env antigenic regions and their variants could therefore help to improve design of preventive HIV vaccines. HIV Envelope protein based immunogens can consist of soluble gp120 monomer (9, 10), or gp140 trimers that may include molecular modifications to stabilize a near-native quaternary structure of the protein (11, 12). In addition, derivates of gp41 alone and gp120 have been used in preclinical HIV vaccine studies (9, 13) to focus the immune response toward these Env regions. Env-based immunogens are also often encoded in recombinant viral, DNA/RNA vaccine vectors either as gp120, gp140 or as membrane-anchored molecular forms of the Env glycoprotein, such as gp160/gp150 (14) allowing expression of the Env immunogens *in vivo* mimicking natural viral infection. HIV vaccination strategies entering clinical testing often include multiple Env immunogen variants and prime-boost regimens that combine different vaccine vectors. These can be administered simultaneously (15) or consecutively (16). The immunologic consequences of such strategies have been poorly defined and methodologies for comprehensive evaluation have been limited so far. For instance, it is unclear whether sequence-heterologous prime-boost vaccination strategies improve or even worsen recognition of highly variable regions, by diluting the “net sum” B-cell receptor stimulus for the hypervariable regions.

The UKHVC003 (UK003) and TaMoVac01 vaccine trials investigated the immunogenicity of a recombinant, trimeric gp140 protein (CN54gp140) in combination with different DNA-MVA prime-boost strategies. During the UK003 trial, vaccine recipients of the standard group (SG) first received a DNA vaccine encoded CN54gp160 and then a secreted form of CN54gp120 encoded in the MVA (17). In contrast, during TMV01, vaccine recipients first received the HIVIS-DNA vaccine that included among others three plasmids encoding for subtypes A, C and B gp160 Env sequences and then two boosts with MVA-CMDR-gp150, which encodes for a membrane-anchored, CRF01_AE derived Env gp150 (16, 18). Both trials included two final boosts with the subtype C derived, trimeric protein CN54gp140 in glucopyranosyl lipid adjuvant (GLA) were administered (19). In order to identify vaccine parameters that influence vaccine-induced recognition of different Env antigenic regions and their variants, we systematically compared the IgG recognition of individual, linear HIV Envelope antigenic regions between the two trials and further mapped the most immunodominant antigenic regions to native trimeric gp160 and monomeric gp120 protein structures.

## Materials and Methods

### Vaccinations and Volunteers

**Figure 1:**
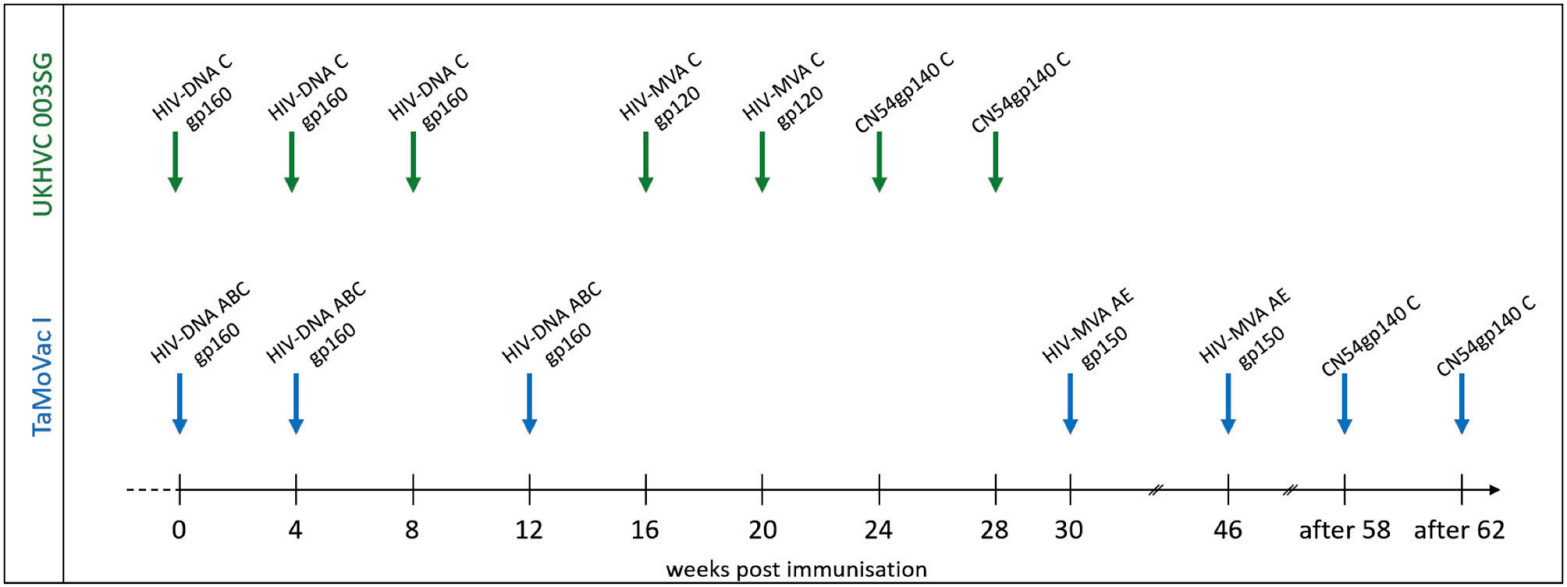
Vaccination schedules in the UKHVC003 Standard Group and TaMoVac01 trial. The **UKHVC 003 SG** vaccine comprised DNA plasmids (8 mg/immunization) and MVA (10^8^ TCID50/immunization) coding for matched subtype C-derived CN54 based Env and ZM96 Gag, Pol, and Nef immunogen sequences. Of note, MVA-C expressed only the gp120 portion of Env. The CN54rgp140 protein (100 ug/immunization) was adjuvanted with 5ug GLA-AF and administered as two additional boosts. The **TaMoVac I** vaccination regimen included DNA vaccination (600 or 1,000 μg/immunization) with 7 plasmids (encoding for Env subtypes A, B, and C and Rev subtype B as well as Gag subtypes A, B, and RTmut subtype B) delivered intradermal, followed by MVA-CMDR (10^8^ pfu/immunization), expressing HIV gp150 (subtype E), Gag and Pol (subtype A), and two additional boosts with 100 μg of CN54rgp140 protein adjuvanted with 5 μg GLA-AF.

Plasma samples from adult vaccine recipients in the TaMoVac01 [TMV01, *n* = 17 (16)] and UKHVC Spoke 03 trials [UK003, *n* = 11 (17)] were analyzed in this study. The UK003 trial was a clinical phase 1 vaccine trials. TMV01 was a phase 2a trial. Neither of these trials tested for vaccine efficacy. As shown in Figure 1, TMV01 and in the UK003SG consisted of three DNA vaccinations, followed by two boosts with a recombinant Modified Vaccinia Ankara virus and two additional boosts with 100 μg of the recombinant subtype C Envelope protein CN54rgp140 adjuvanted with 5 μg of the synthetic Toll like receptor 4 agonist GLA-AF. The recombinant CN54gp140 Env protein is an uncleaved, soluble trimer that does not contain stabilization mutations, such as SOSIP (16).

During the TMV01 trial, healthy volunteers from Tanzania received seven TMV01-DNA plasmids (total amount between 0.6 and 1.0 mg/vaccination, referred to as HIVIS-DNA in other publications) at weeks 0, 4, and 12 intradermally using the Zetajet device. While these subjects belonged to three different randomization groups, there was no difference in gp160-specific IgG titer between these groups (20). Three of the seven plasmids encoded for gp160 of HIV-1 subtypes A, B, and C. The other four plasmids encoded for HIV-1 Rev and Gag (subtype B), Gag (subtype A) and a mutated form of the Reverse Transcriptase (subtype B) (21). At weeks 30 and 46, two boosts with 10^8^ PFU HIV-MVA-CMDR-gp150, expressing a membrane-anchored gp150 CRF01_AE, were given intramuscularly. 12 or above weeks after the second MVA-CMDR boost, two additional boosts of 100 μg of CN54rgp140/5 μg GLA were administered into the deltoid muscle of the left arm.

The UK003SG included healthy volunteers from the United Kingdom. The DNA vaccination consisted of two DNA plasmids; one encoded (CN54gp140) Env and the other a (ZM96) Gag-Pol-Nef fusion protein (17). Four milligrams of each DNA plasmid was administered intramuscularly (IM) at weeks 0, 4, and 8 followed by two IM boosts with 10^8^ TCID50 of the MVA-C-gp120 at weeks 16 and 20 and two additional IM boosts with 100 μg CN54gp140/5 μg GLA-AF at weeks 24 and 28. The MVA-C-gp120 expressed a secreted form of CN54gp120 Env and Gag-Pol-Nef polyprotein from two back-to-back synthetic early/late transcriptional promoters (22, 23).

The study documents for both trials were reviewed and approved by the relevant Ethical review boards and participants of both trials gave fully informed written consent according to the Declaration of Helsinki before any study procedures were conducted. The TaMoVac01 trial is registered at the World Health Organization International Clinical Trials Registry with registration number PACTR2010050002122368. The UKHVC Spoke 03 trial was registered with the European Union Drug Regulating Authorities for Clinical Trials (EUDRACT TC 2012-003277-26) and Clinical Trials.gov (NCT01922284) and with the UK Clinical Trials Research Network (UKRN-14173).

### Peptide Array Design

**Figure 2:**
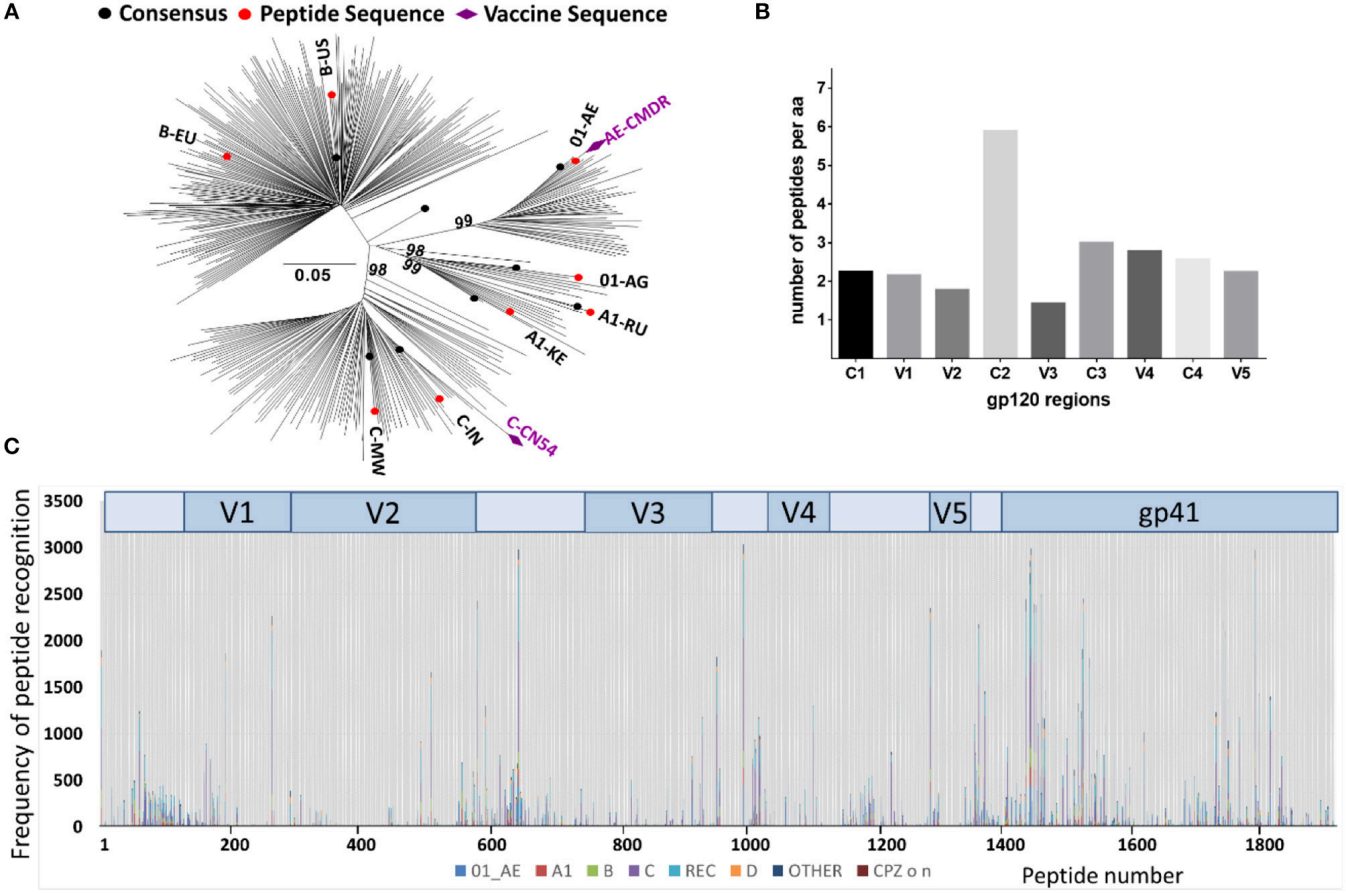
Peptide array design to cover recently transmitted global HIV strains. **(A)** Phylogenetic analysis of acute infection sequences (*n* = 350) from the Los Alamos database used for the selection of the eight isolates (in red) to design the peptide array. Purple diamonds indicate the isolates of the vaccine immunogens CMDR and CN54. Evolutionary analyses were conducted in MEGA6 (24) and the evolutionary history was inferred using the Neighbor-Joining method (25). The percentage of replicate trees (Bootstrap test 100 replicates) in which the associated taxa clustered together is shown above the branches (26). **(B)** Number of 15-mer amino acid (aa) sequences represented in the peptide array. The peptide array was aligned to the HxB2 sequence that was scanned with a sliding window of 11 aa using a three aa step. The Y-axis indicates the number of 11-mer aa sequences found in the array corresponding to a given 11-mer HxB2 window. **(C)** Peptide recognition amongst the known Los Alamos database HIV-1 sequences. 2,078 peptides produced 471,597 complete homology hits amongst 10,956 virus isolates. The Y-axis indicates the number of hits per peptide position, each subtype represented by a different color.

The array consisted of triplicates of 2,212 immobilized 15 mer peptides. Full length Env immunogen sequences included were, CN54gp140 (subtype C) and CMDR (subtype AE) and 8 additional sequences from acute phase primary HIV isolates of subtypes A, C, B, CRF01_AE, and CRF02_AG to maximize coverage of global HIV diversity. These were selected on the basis of acute infection sequences, extracted from the Los Alamos data base (*n* = 6,467). Of those, one predominant sequence from each patient *n* = 350) was retained. After phylogenetic analysis of these 350 acute infection sequences (Figure 2A), we selected eight sequences according the geographic provenance and to the available patient information on Fiebig stage, day post-seroconversion and viral load to insure they were as close from seroconversion as possible in addition to representing the major subtypes (Figure 2A); [06.RU.SP.R163.IorII_13 (subtype A1, Russia, GU481385), Q842.d12.PNS70d.KE (subtype A1, Kenya, AF407160), PHI374_FR (subtype B, France, AF041133), US.1990.BORId9.2F8 (subtype B, USA, EU576282), 25925.2.IN (subtype C, India, EF117273), MW.2003.CHV0011210.0393.C3 (subtype C, Malawi, FJ444215), FR.1996.PHI426 (CRF01_AE, France, AY231158) and PHI127 (CRF02_AG, France, AY231152)] (Supplementary Table 1). Previously defined antigenic regions, frequently recognized by vaccinees in the TaMoVac01 trial (unpublished results), RV144, VAX003, and VAX004 (4), were covered with up to 86 additional peptide variants in hypervariable regions. These were located in the V2 region (peptide starting with HxB2_163), in the V3 region [V3a (HxB2_300-324) and V3b (HxB2_349)], in the C-terminal of V4 (HxB2_409-447), in the gp41 Immunodominant region (HxB2_576-614) and the gp41 cytoplasmic tail (HxB2_696-730). The relative peptide number per envelope region is given in Figure 2B. The final peptide selection was blasted against all published sequences from the Los Alamos data base to establish the per peptide recognition frequency, illustrated in Figure 2C.

### Linear Peptide Array Mapping

Microarrays were processed according to the manufacturers instructions with minor modifications (www.jpt.com). Briefly, the slides were incubated with T20 blocking buffer (Thermofisher) for 10 min. Plasma samples were then added at a dilution of 1:100 in T20 blocking buffer, and incubated for 2 h at room temperature with gentle shaking before washing 5 times with 2.5 ml TBS-Tween (0.5% Tween). The secondary mouse anti-human-IgG Dylight649 (JPT) was then incubated at room temperature for 1 h at a dilution of 1:5,000 in T20 blocking buffer. After 5 washings with 2.5 ml TBS-Tween, and 5 washes with double distilled de-ionized water, the slides were left to dry under a laminar flow hood. Samples from all time points from one individual were processed simultaneously. Slides were scanned on a GenePix 4000A scanner and processed using GenepixPro 6.0 software at 650 and 532 nm to generate a Tiff image file. The array lay out was then added using an array-specific.gal file. Accuracy of the array alignment was controlled, and individual features were adjusted or excluded manually when needed during the quality control step. Individual peptide-specific IgG responses were then mapped after subtraction of FI values from baseline plasma. Positive responses were defined as equal or above 2,500 fluorescence intensity units after subtraction of the pre-vaccination value.

### R Script Based Analyses of Peptide Variant Recognition by IgG in the Context of Peptide Sequence Phylogeny and Frequency of Occurrence

A R-script was developed to visualize the magnitude of the IgG recognition of peptide variants of a given antigenic region, and linking these data to the phylogenetic relationship of these sequences as well as the frequency of their occurrence in the database. Our script utilizes the R package GGTREE by Yu et al. as a base (27). The new script uses the existing newik and fasta files and combines them within a single step into a new form of result visualization in the context of the magnitude of IgG recognition (color coded) and the frequency of occurrence (coded by icon size, HIV database) of a given peptide variant. For our analyses, a maximum likelihood phylogenetic tree for 36 antigenic variants of the HIV V3 Tip region (HxB304) was generated after sequence of 75 amino acids was added as scaffolding to each of the 15 mer peptides. The tree file was then converted to a newik file for input into the R-script analyses. To interrogate the frequency of variant sequence occurrence, more than 3,500 representative HIV primary isolate whole length Envelope sequences were selected from the global HIV sequence database (www.hiv.lanl.gov) using following parameters: uniqueness, one sequence per patient and an overall good representation of the global HIV epidemic and saved as a fasta file. The subtype distribution and numbers of subtype-specific sequences in these selected sequences were as follows: subtype B (*n* = 1,459), subtype C (*n* = 969), CRF01_AE (*n* = 419), subtype A (*n* = 205), recombinant molecular forms of subtype B and C (*n* = 156), recombinant molecular forms of subtypes A other than CRF02_AG (*n* = 138), all other molecular forms combined (*n* = 340).

## Results

### Basic Demographics of Subjects Included in the Peptide Array Analyses

Analyzed TMV01 vaccine recipients (*n* = 17) were all black Africans, had a median age of 29 years (range 18–38) and included 7 females. The 11 vaccine recipients from UK003SG included in the analyses (all made available for peptide array mapping) were all white Europeans and had a median age of 36 years (range 20–44) and included 5 females.

### IgG Recognition of Linear Antigenic Regions Envelope After the Second MVA Boost

**Figure 3:**
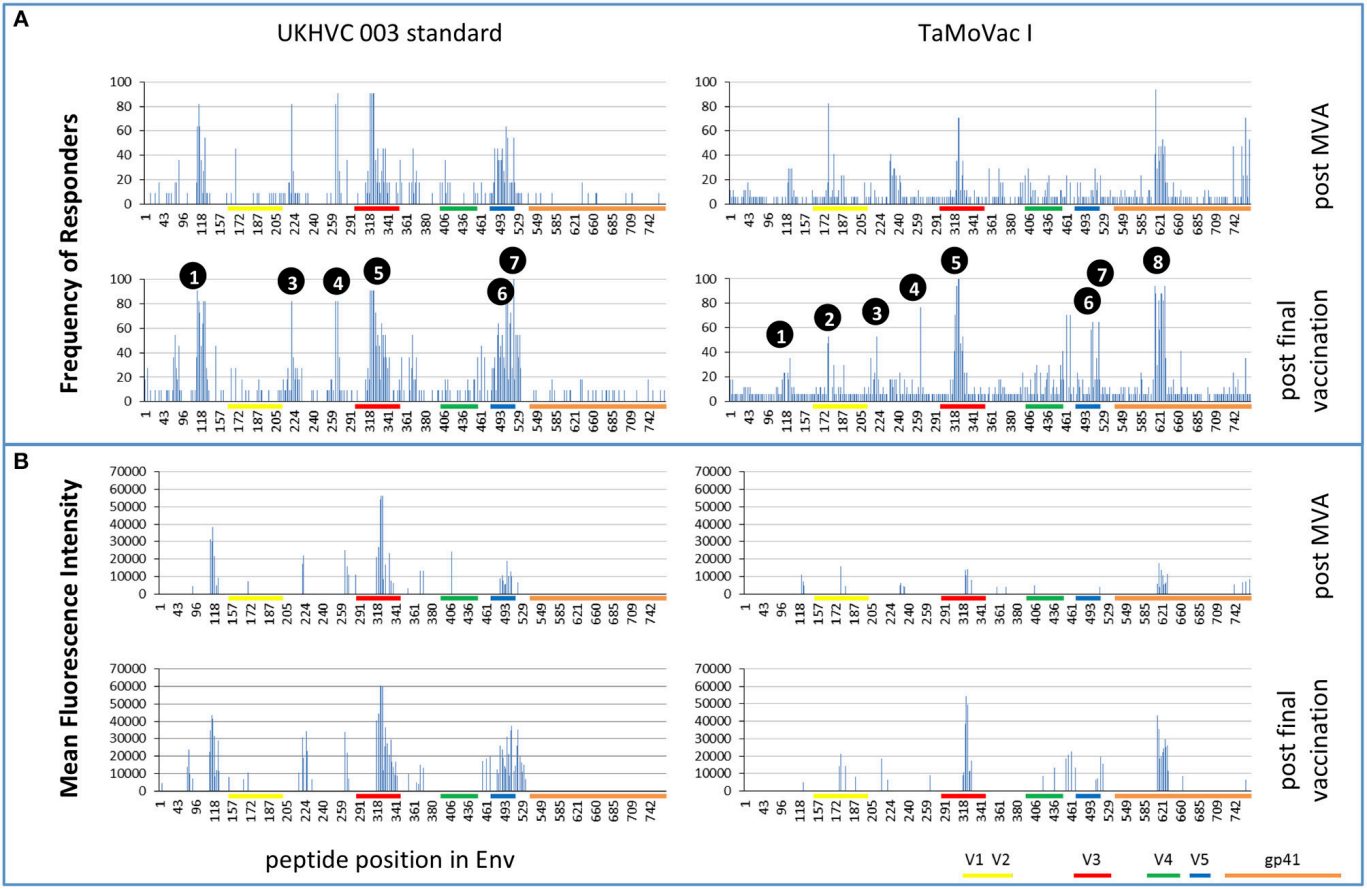
Mapping of antigenic regions targeted by Env-specific IgG responses in UKHVC003 and TaMoVac01. The frequency of responders **(**FOR, y-axis) to antigenic regions within the Envelope protein **(A)** after the second MVA (first row) and after CN54rgp140/GLA boosts (second row) is shown for the UKHVC_003 standard group (left panels, *n* = 11), and for TaMoVac01 recipients (right panels, *n* = 17). Immunodominant regions 1–8 are indicated. For region-specific IgG responses occurring in at least 25% of vaccinees, the mean fluorescence intensity (MFI) is shown in **(B)** after MVA (third row) and after CN54rgp140/GLA boosts (fourth row) for the two groups. IgG responses against individual antigenic regions were considered positive, if the corresponding fluorescence intensity was above 2,500 after subtraction of the pre-vaccination value. Mapping results from UKHVC003 were partially published previously (17).

**Figure 4:**
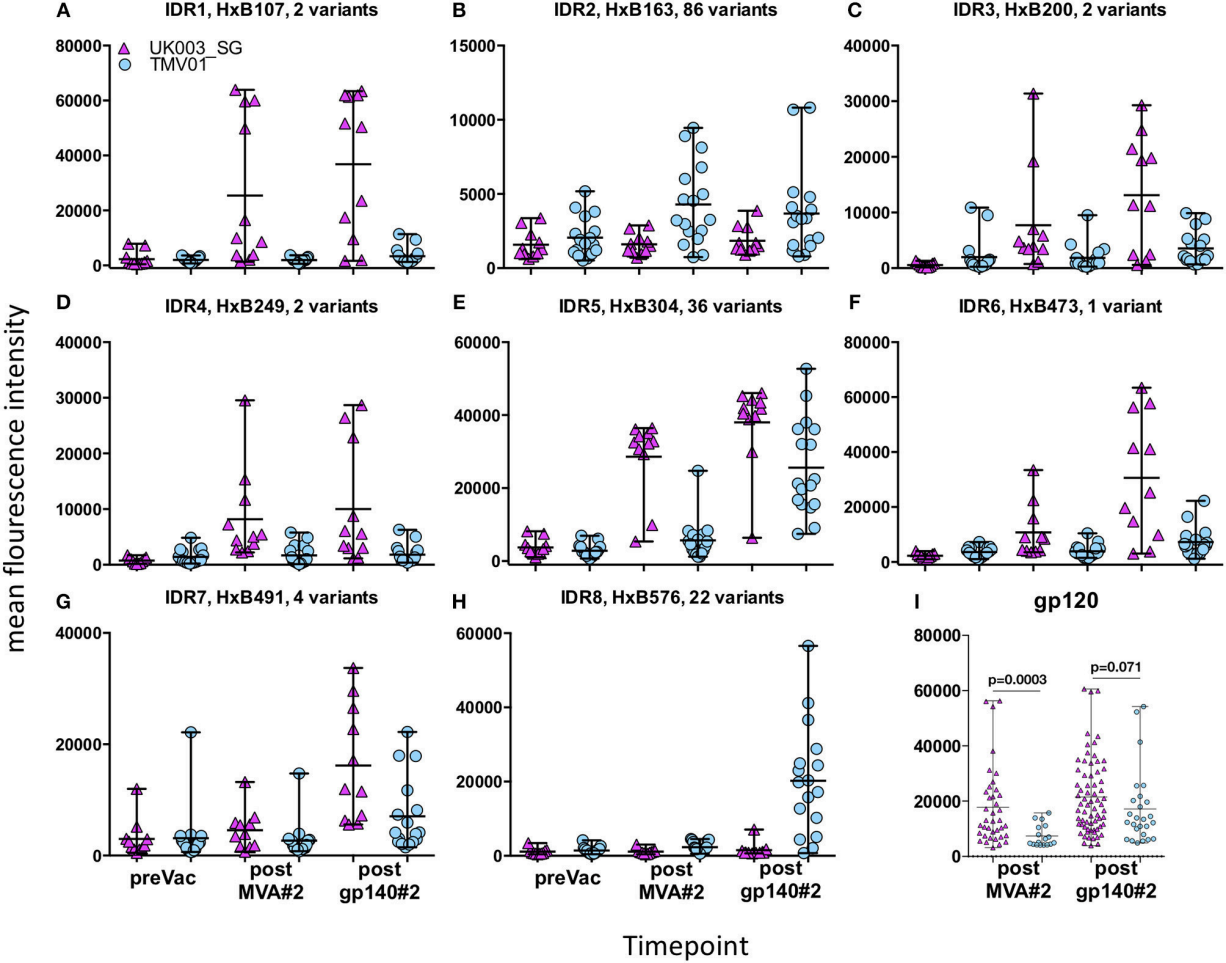
Comparison of IgG responses targeting selected frequently recognized regions in UK003SG or TMV01. Shown is for the eight selected immunodominant regions [IDR1-8 **(A–H)**] for each analyzed vaccinee the mean recognition MFI over all tested antigenic variants at baseline, after the second boost with MVA and after the second boost with CN54gp140/GLA. The first HxB aa position of the 15 mer peptide and number of tested variants are indicated for each antigenic region. The mean MFI of responses for each antigenic region recognized within gp120 by above 25% of vaccinees are shown in **(I)**. The results of the statistical analyses by Mann-Whitey test comparing data from the two trials are indicated only for **(I)**. Data points from UK003SG and TMV01 are shown in magenta and light blue, respectively. The statistical analyses for differences in mean recognition magnitude for **(A–H)** is provided in Table 1.

Four weeks after the second MVA boost, we observed profound differences in the antigenic regions targeted by the Env-specific IgG response between UK003SG and TMV01 (Figure 3; Table 1). In total, 8 immunodominant regions (IDR, defined by a frequency of responders (FOR) of >50%, numbered black balls in Figure 3) were detected considering both trials. For 7 of these 8 regions the magnitude of peptide recognition (Mean Fluorescence Intensity (MFI) of all tested peptides for a given region) differed significantly between UK003SG and TMV01 vaccine recipients (all *p* < 0.01, Table 1, Figures 4A–H). Recipients of the UK003SG regimen exclusively targeted antigenic regions located within gp120, including the constant region (C) 1 [starting at amino acid (aa) positions 112 (IDR1, Mean Fluorescence Intensity (MFI): 25,412)], in the C2 region (aa221, IDR3, MFI: 7,702, and aa270, IDR4, MFI: 8,176) and two regions in C5 (aa503, IDR6, MFI: 10,800, and aa491 (IDR7, MFI: 4,540). Corresponding HxB2 locations are provided in Table 1. None of these regions were recognized by more than 2 of 17 TMV01 vaccine recipients. Participants in both trials targeted the V3 loop tip (IDR5, aa322–326), but we observed a 5-fold higher MFI for the UK003SG as compared to for TMV01 vaccine recipients. In contrast to UK003SG, TMV01 vaccine recipients frequently targeted the V2 loop (IDR2—aa163, MFI: 4,300, *p* = 0.003) and an immunodominant region within gp41 (IDR8—aa608, MFI: 2,336, *p* = 0.005). Of note, gp41 was not contained in MVA-C-gp120 used in UK003SG. Considering all gp160 regions that were targeted by at least 25% of vaccine recipients in either trial [UK003SG (38 regions) as compared to TM01 vaccine recipients (30 regions)], mean magnitudes for IgG recognition of antigenic regions was higher in UK003SG as compared to TMV01 (mean MFI: 17,823 vs. 7,791, *p* < 0.0001, data not shown). Similarly, considering only IgG recognition of gp120, a 2.2-fold broader response was detected in UKHVC003S vaccine recipients (38 vs. 17 regions targeted by ≥25% of vaccine recipients, Figure 4I) and individual region-specific IgG responses had higher mean magnitudes in UK003S as compared to TM01 vaccine recipients (mean MFI: 17,823 vs. 7,410, *p* = 0.0003). Together these data demonstrate that the largely sequence homologous UK003SG regimen induced a stronger and broader IgG response, with little overlap in frequently recognized Env regions after the second MVA boost as compared to the sequence heterologous TM01 regimen.

**Table 1:** Selected frequently recognized HIV Envelope regions and their recognition after the second MVA and after the second CN54gp140 vaccination.

					**Post-MVA#2-MFI of all subjects**			**Post-CN54gp140#2-MFI of all subjects**		
**IDR**	**aa**	**Representative sequence**	**HxB2**	**Env. region**	**UK003 SG**	**TMV01**	***p*-Value**	**UK003 SG**	**TMV01**	***p*-Value**
1	112	DIISLWDQSLKPCVK	107–121	C1	25,412	1,936	<0.0001	36,822	3,384	0.0004
2	176	ELRDKKQKVHALFYK	163–177	V2	1,604	4,300	0.0029	1,848	3,681	0.0530
3	221	AITQACPKVTFDPIP	200–214	C2	7,702	1,855	0.0024	13,108	3,565	0.0220
4	270	HGIKPVVSTQLLLNG	249–263	C2	8,176	1,692	0.0004	10,033	1,805	0.0006
5.1	322	NNTRKSIRIGPGQTF	301–315	V3 Tip	11,403	2,193	<0.0001	19,514	6,852	0.0005
5.2	325	RKSIRIGPGSTFYAT	304–319	V3 Tip	28,606	5,669	<0.0001	38,003	25,618	0.0126
6	503	GDMRNNWRSELYKYK	473–487	V5C5	10,800	3,902	0.0041	30,591	7,271	0.0035
7	521	IKPLGVAPTTTKRRV	491–505	C-term gp120	4,540	2,684	0.0590	16,193	7,044	0.0058
8	608	LQARVLAVERYLKDQ	576–591	gp41	1,104	2,336	0.0049	1,544	20,221	<0.0001

### IgG Recognition of Linear Antigenic Env Regions After the Second Immunization Boost With CN54gp140/GLA

The pattern of Env recognition still differed between the two groups at 4 weeks post-final vaccination with the second CN54gp140/GLA. Mean magnitude of recognition was still significantly higher in UKHVC_003 vaccinees in 6 of 8 IDR regions (Table 1; Figures 4A–H) and the magnitude of recognition still differed significantly for the same IDRs as after the second MVA boost (*p* < 0.05). However, more TMV01 vaccine recipients were now also recognizing most of the 8 IDRs including the V3 region and other more conserved antigenic regions within gp120. Of note, there was no measurable effect of the CN54gp140 protein boost on IgG recognition of the V2 loop in either group. Three N-terminal antigenic regions in C1 of gp120 (HxB74, 104–109, 117–121, Figure 3) were still recognized exclusively by UKHVC_003 participants. Consecutive peptides covering the tip of the V3 region (located at HxB aa312–315) were targeted by >90% vaccinees with a high MFI, of max. 38,003 and 25,618 in UK003SG and TMV01, respectively. Interestingly, recognition of the IDR8, covering part of the immunodominant region of gp41, was boosted by CN54rgp140 administration in TMV01 participants, while this region was not being recognized to detectable levels in UK003SG (*p* < 0.0001). This suggests that although CN54gp140 contains the extracellular domain of gp41, the region-specific immunogenicity was not sufficient to mount substantial levels of gp41 specific ABs in the absence of MVA mediated priming. Considering all antigenic regions within gp120, a broader Env-specific IgG response was detected after the second CN54gp140/GLA boosts in vaccine recipients of both trials. UK003SG recipients had a 2.6-fold broader recognition (68 vs. 26 regions targeted by ≥25% of vaccine recipients) compared to TMV01 (Figure 4B). Individual region-specific IgG responses had higher mean magnitudes in UK003SG as compared to TM01 vaccine recipients.

### Assessment of Immunogen Sequences, Protein Structure and Glycan Profiles in the Eight Most Immunodominant Regions

**Figure 5:**
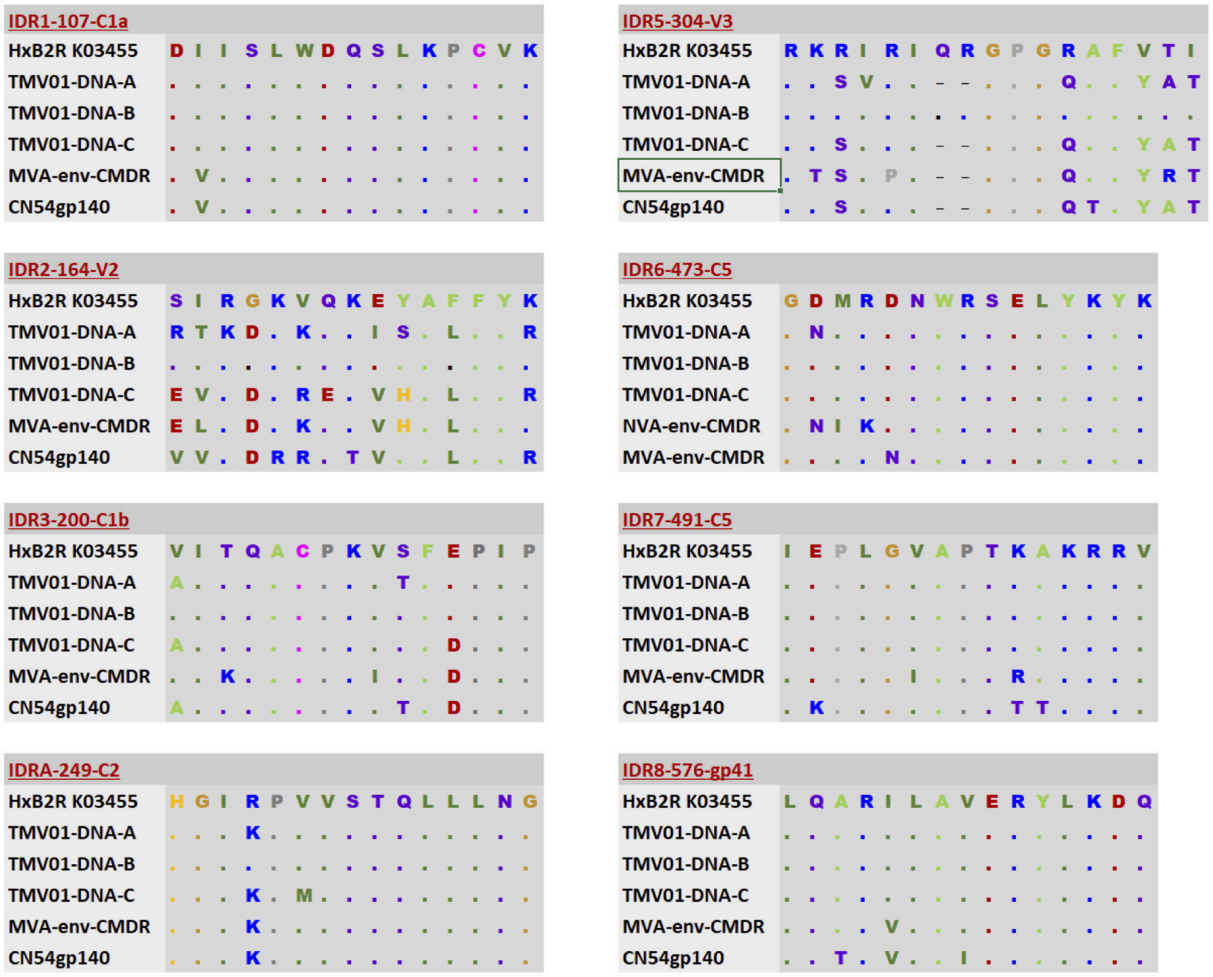
Alignment of immunogen sequences for the eight selected immunodominant antigenic regions for immunogens included in TMV01 and UK003SG. From top to bottom; the first row indicates the Immunodominant region, HxB2 location and Region name and shows a representative sequence for this region. Row 2–4 show three sequence variants of the TMV01-DNA encoded Env proteins representing subtypes A, B, and C, respectively. Row 5 shows the MVA-CMDR-gp150 encoded subtype E sequence that was used during TMV01. Row 6 shows the CN54 sequence, which matches the DNA and MVA-C-gp120 encoded Env immunogens of UK003SG and the CN54gp140 recombinant protein administered in both trials.

Differences in the Env immunogen sequences during the prime-boost vaccinations might have contributed to the differences observed here in vaccine-induced IgG recognition of HIV Env. Figure 5 shows an alignment of the immunogen sequences for each of the 8 immunodominant antigenic regions. UK003SG participants received CN54 based immunogen sequences that were identical in gp120 but deleted of gp41 in the MVA-C-gp120. The TMV01 vaccination contained much more diverse Env immunogen sequences for most of the IDRs. The IDR 8 within gp41 region was included by the TMV01-DNA gp160 encoding plasmids, MVA-CMDR-gp150 and in the CN54gp140 boost.

**Figure 6:**
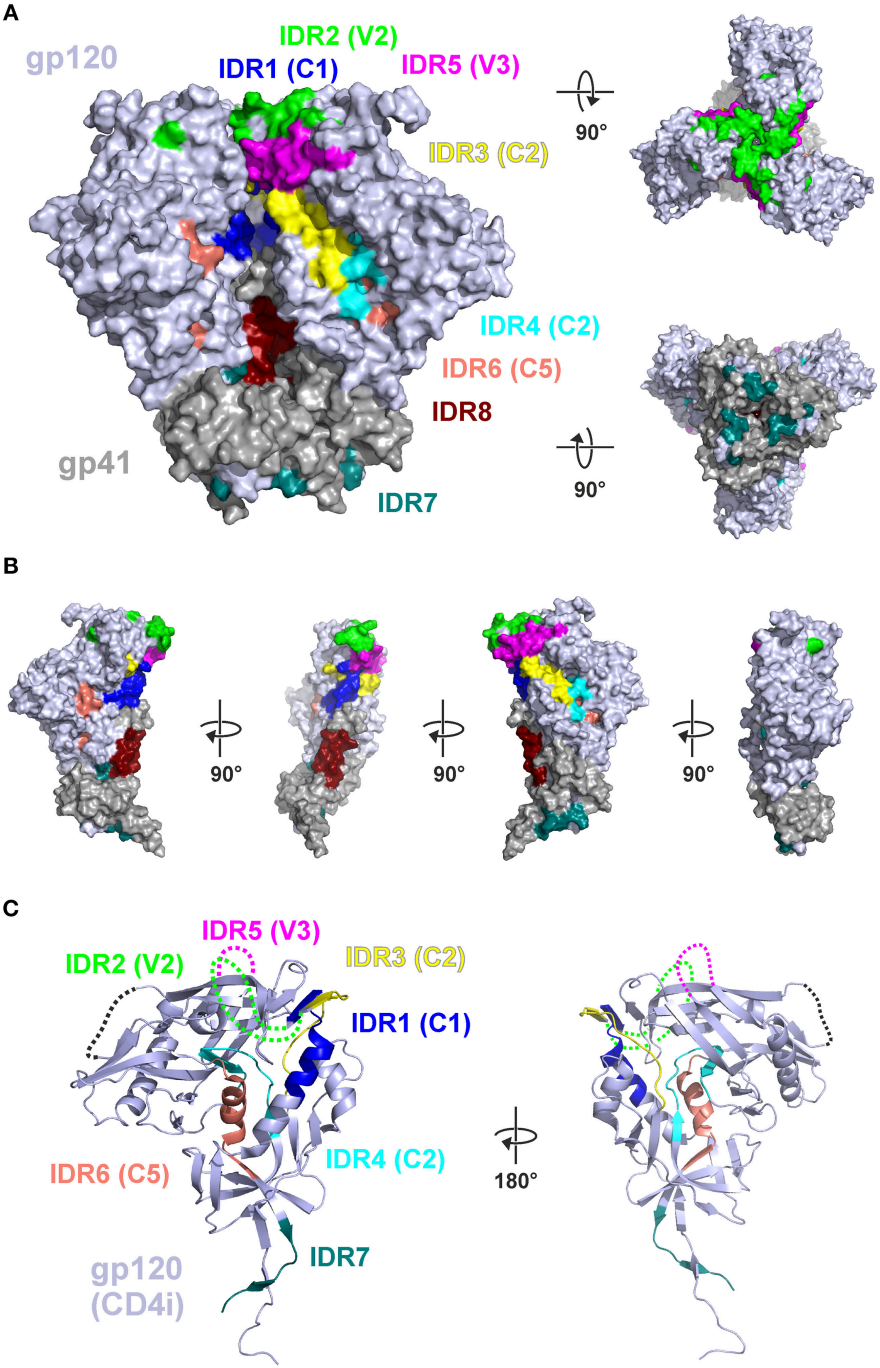
Mapping of immunodominant peptide regions (IDR) to the structure of Env. **(A)** Color-coded solvent accessible surface area (SASA) of the eight IDRs projected on a native like Env trimer (C-clade consensus structure, PDB ID 8ck9). Glycans were removed and IDRs are highlighted in all protomers. Both IDR5.1 and IDR5.2 are combined as IDR5. **(B)** Eight IDRs projected onto the surface of a gp120-gp41 protomer from **(A)**. **(C)** Location of the IDRs in the context of a CD4-induced (CD4i) gp120 structure (PDB ID 3jwd). Variable loops 1–4 which are deleted or not resolved in the structure are symbolized by dotted lines.

Protein structural and conformational aspects as well as glycosylation patterns might also influence the magnitude of IgG recognition of individual Env regions. Figure 6 shows the 8 IDRs as color coded solvent accessible surface areas (SASA) on a native trimeric Env protein structure (here: stabilized C-clade consensus structure (28) and in a gp120 structure in the CD4 induced state [HXBc2 gp120 core structure (29)] as this is a possible alternative conformation present *in vivo* during immunizations.

In this well-ordered trimer structure IDR2 (V2) and IDR5 (V3) are oriented toward the trimer apex. Most of other IDRs are either located in the inter-gp120 interface in the direction of the trimer axis [IDR1 (C1), IDR3 (C2)], at the gp41-gp120interface (IDR7) located in gp41 in the direction of the trimer axis (IDR8) (compare Figure 6B). IDR4 and IDR6 are located in the core of gp120, mostly buried under the outer domain. Hence, in a well-ordered trimer structure, the majority of these epitopes are potentially concealed from antibody recognition. In contrast the context of gp120 (Figure 6C) most of the gp120 IDRs are well-accessible except IDR4 and IDR6, which are still buried in the protein core.

We next analyzed glycosylation sequence motifs of the 8 most immunodominant regions using the HIV sequence compendium 2015—HIV-1 proteins (Table 2). Six of the eight IDRs did not contain any glycosylation motif. IDR4 ended in such a motif and IDR5.1 (V3) started with such a motif. Glycosylation motifs were located in very close vicinity (≤5 aa) upstream of 5 IDRs. Glycosylation motifs downstream were located in varying distance (0 to >20 aa). Together our results suggest that non-glycosylated Env regions located at the Envelope tip (V2 and V3) or in the inter-gp120-interface are the most immunodominant regions of gp120 as detected by linear peptide array analyses.

**Table 2:** Analyses of Envelope protein glycosylation motifs within or near frequently recognized immunodominant regions.

				**Glycosylation motifs**			
**IDR**	**Representative sequence**	**HxB2**	**Env. region**	**Inside region**	**Downstream (N-terminal)**	**Upstream C-terminal**	**Other comments**
1	DIISLWDQSLKPCVK	107–121	C1	None	16 aa	10 aa	2 aa from CD4 BS residue
2	ELRDKKQKVHALFYK	163–177	V2	None	2 aa	5 aa	–
3	AITQACPKVTFDPIP	200–214	C2	None	1 aa	14 aa	–
4	HGIKPVVSTQLLLNG	249–263	C2	C-terminal	5 aa		–
5.1	NNTRKSIRIGPGQTF	301–315	V3 Tip	N-terminal		13 aa	–
5.2	RKSIRIGPGSTFYAT	304–319	V3 Tip	None	2 aa	17 aa	–
6	GDMRNNWRSELYKYK	473–487	V5C5	None	7 aa	>20 aa	Contains CD4BS residues
7	IKPLGVAPTTTKRRV	491–505	C-term gp120	None	5 aa	>20 aa	6 aa from gp120 C-term
8	LQARVLAVERYLKDQ	576–591	gp41	None	>20 aa	20 aa	Ends in gp41 IDR

### Comparison of Antigen Variant Recognition Within V3 and V2

**Figure 7:**
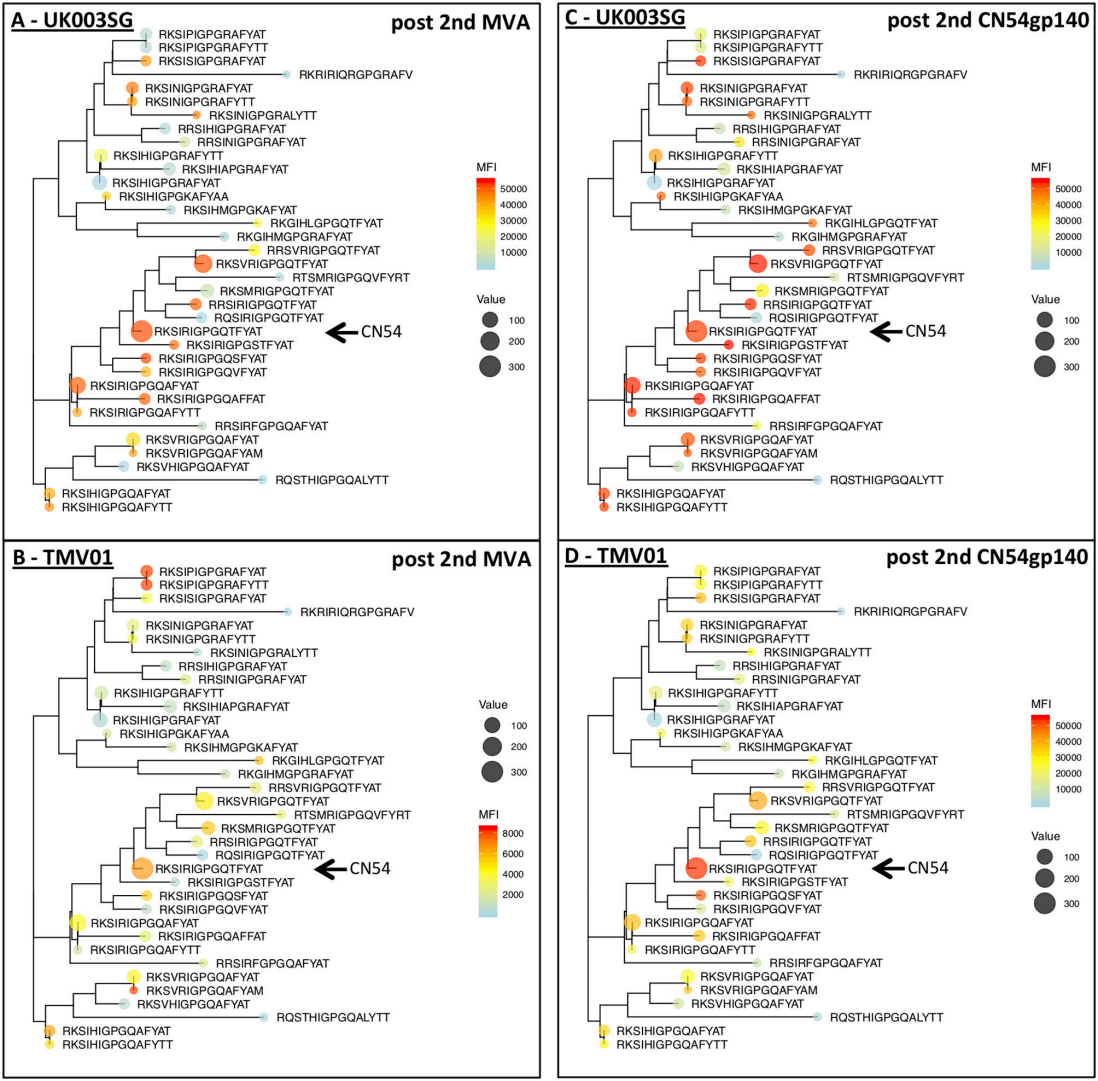
Phylogenetic heat map analyses of IgG recognition of the Env variable region 3 in UK003SG and TMV01 vaccine recipients. Shown is a heat map of mean signal intensities of IgG recognition of 36 peptide sequence variants of the variable region 3 tip (HxB304_ RKSIRIGPGSTFYAT) in the context of their phylogenetic relationship and their frequency of occurrence in the HIV database represented by the icon size. **(A,B)** Show the results after the second MVA boost, **(C,D)** for the results after the second CN54gp140/GLA boost with the vaccine. A maximum likelihood phylogenetic tree for 36 antigenic variants of the HIV V3 Tip region was generated after sequence of 75 amino acids was added as scaffolding to each of the 15 mer peptides represented on the peptide array. Please note the different heat map scale in **(B)**. The arrows point to the CN54 isolate sequence in the respective trees.

The V3 region was among the most immunodominant regions in gp120 after the CN54gp140 protein boost in both vaccine trials with particularly strong recognition of the V3-tip region (HxB2 aa position 301–320). Cross-recognition of the V3-tip region was assessed with >35 antigenic variants for each of the overlapping peptide regions HxB301–316 and HxB304–318. In the UKHVC003SG, strong IgG recognition of many different antigenic variants of the V3 tip was already detected after the two MVA-CN54gp120 boosts (Figure 7A, not shown for HxB301–316), whereas IgG recognition was much weaker after the two MVA-CMDR-gp150 boosts in the TMV01 trial (Figure 7B). The numeric MFI data for V3 peptide variant recognition shown on the phylogenetic heat map trees are provided in Supplementary Table 2. Of note, relative recognition of the same peptide variant after the two MVA boosts was quite similar between both trials. One example, V3-tip sequence variants RKSIRIGPGQTFYAT (CN54 sequence, arrow in Figure 7) and the related RKS***V***RIGPGQTFYAT were recognized in both trials. Both of these sequence variants are overrepresented in the HIV sequence database. The most notable difference between the two trials is 1.) the much higher magnitude of recognition for almost all recognized V3 tip variants in the UKHVC003 trial and 2.) the comparatively good recognition of the two related peptide variants RKS***IP***IGPG***RA***FY(A/T)T upon MVA-CMDR vaccination in TMV01 participants, with a proline in position 5, which is also contained in the immunogen CMDR sequence. In summary these results show that the use of diverse immunogen sequences in prime and boost during the TMV01 trial was not associated with better cross-recognition of V3-tip variants as compared to the use of a single V3 immunogen sequence during UKHVC03.

By contrast, the V2 region was targeted exclusively by TaMoVac 01 vaccinees, in which V2-specific responses peaked after the second MVA-CMDR. The response was focused on a single peptide region (HxB163/164) without measurable recognition of the adjacent overlapping regions. Out of the 86 variants tested, <10 sequence variants were recognized well by the majority of TMV01 vaccinees after the second MVA-CMDR (Supplementary Figure 1). The numeric MFI data for V2 peptide variant recognition shown on the phylogenetic heat map trees are provided in Supplementary Table 3. Most of the well-recognized variants were closely related to the MVA-CMDR-gp150 encoded immunogen sequence (≤2 mutations). Administration of CN54gp140/GLA did not boost these responses (data not shown). Both TMV01-DNA encoded Env during TMV01 as well as the CN54 immunogen sequences have 4–7 aa mismatches in this 15 mer region compared to the MVA-CMDR sequence (Figure 5), raising the question whether the DNA vaccine primed this V2-specific response.

## Discussion

A better understanding of vaccine parameters influencing IgG recognition of individual Env regions and their antigenic variants can contribute to rational HIV vaccine design. Using a linear peptide array approach, we compared Env-specific IgG responses in two clinical trials, which tested different DNA prime MVA boost vaccine combinations followed by additional boosting with the same recombinant, trimeric CN54gp140 protein. All Env immunogen sequences in UK003SG were derived from HIV subtype C isolate CN54 including a MVA vector encoding for a secreted molecular form of gp120 (17). In contrast, TMV01, Env immunogen sequences were multivalent and included a MVA vector encoding for a membrane-anchored, functional molecular form of gp150 (18). The functionality of this gp150 protein has been determined by its capacity for induction CD4- and CCR5-dependent cell fusion *in vitro* (18). The two groups also differed in the exact timing of immunogen administrations, the route and amount of DNA administration, trial location and ethnicity/race of participants. While we consider it unlikely, we cannot exclude the possibility that some of these differences may also have influenced the pattern of Env-specific linear epitope recognition by IgG. Here, we focus the discussion on the potential impact of immunogen sequence variability and structure on the pattern of vaccination-induced Env-specific IgG recognition.

The pattern of Env epitope recognition detected UK003SG after the MVA boosts, shared many features with those described previously for vaccine trials Rv144, Vax003 and Vax004 (4). The IDR1 (HxB104) was frequently recognized in UK003SG recipients, Rv144 and Vax003 (“C1a”) and to a lesser degree in Vax004. UK003SG and Vax003 recipients frequently recognized C1b (HxB120). Further, similar regions in V3 and C5 were recognized in UK003SG after the MVA-C-gp120 boosts and after final vaccination in Rv144, Vax003 and Vax004, which were all based on gp120 monomeric Env. Some of these targeted regions differ from those targeted during natural HIV-1 infection [(4) and unpublished observation], and significantly differed from those frequently targeted after the MVA-CMDR-gp150 boosts in TMV01. The usage of gp120-based immunogens in these trials may therefore induce a different Env recognition pattern as compared to the membrane-bound, functional Envelope protein encoded in the MVA-CMDR-gp150. Indeed, the induction of Abs binding to Env epitopes located in the inter-gp120 interface that may be structurally shielded in a native trimer might be direct consequence of soluble gp120 secreted from cells infected with HIV-MVA-gp120 (UK003SG), where these regions are readily accessible to antibodies (compare Figure 6). While speculative, such structural and steric features may also explain why targeting of these epitopes within the relatively conserved inter-gp120 interface was not associated with immune protection during previous efficiency trials Rv144, Vax003, or Vax004 (4).

The magnitude of recognition of most gp120 IDRs was comparatively weak in the TMV01 participants, which contrasted the strong recognition of these regions in UK003SG. The comparatively low degree of sequence conservation between prime and boosts might also have contributed to this pattern; sequence conservation is 100% for UK003SG and always less for TMV01 vaccine regimen. IDR1 (“C1a”) or IDR7 (“C5a”) sequences however were well-conserved between the TMV01 DNA and MVA immunogens. Non-recognition after the MVA boosts of these regions can therefore not be explained by sequence differences in the TMV01 immunogen composition. As mentioned above, the encoded immunogens of MVA-CMDR-gp150 and the MVA-C-gp120 differ significantly in structural features that are relevant for epitope accessibility to antibodies; particularly IDR7 (“C5a”) is in spatial proximity with gp41 and contains amino acids in direct contact to gp41 (29). Hence, IDR7 may be accessible only on gp120 based immunogens, but much less so on the membrane-anchored, functionally intact Env protein encoded by MVA-CMDR-gp150. As shown in Figure 6, IDR7/C5a is most probably occluded on the functional trimer and on soluble trimers made of SOSIP gp140 (11, 29, 30). However, CN54gp140 vaccination boosted IDR7/C5a-specific IgG responses in UK003SG, while repetitive vaccination with CN54gp140 protein alone only induced weak recognition of C5a (31). Negative-stain electron microscopy analyses suggested that CN54gp140 is an aberrant trimer with partial disintegration into gp120 and gp41-ectodomains (12). Hence, C5a may be *partially* accessible on the CN54gp140 immunogen. Besides structural occlusion and sequence characteristics, glycosylation of epitopes may abrogate recognition of the corresponding non-glycosylated peptides. Roughly 90 N-linked glycans−28 per protomer—shield the protein surface of native, trimeric Env from immune recognition. N-linked glycan compositions have been characterized quite detailed and mapped to the trimeric Env protein structure previously (32, 33). Almost all detected IDRs in this study did not contain such glycosylation motifs, except for two IDRs, which start started or ended in such a motif (IDR5.1, IDR4). Close vicinity to glycosylated sites was therefore not abrogating IgG recognition of IDRs in our study. High glycan shielding of the outer trimer surface and/or missing glycans in the peptide array might be a reason for its comparatively lower immunogeneicity and/or low reactivity of the corresponding non-glycosylated peptides in the micro array assay. Interestingly, recombinant, monomeric gp120 has increased levels of N-linked complex-type glycans, but decreased glycan occupancy in 10 of the 24 sites compared to trimeric molecular forms of Env (33). It is one important limitation of current peptide array approaches that these do not allow to interrogate the influence of natural N-linked glycans on linear epitope recognition by binding antibodies. Incorporation of natural Env glycosylation in synthetic peptide arrays would therefore be useful to better define their influence on linear epitope recognition. In follow-up libraries this could be considered [e.g., for N-linked (GlcNAc…) at Asn residues]. However, limited availability of such building blocks and high cost hamper a comprehensive characterization of the influence of glycosylation on antibody recognition during peptide array analyses. In summary, sequence-, structural-, and glycan related differences between Env immunogens used in UK003 and TMV01 most likely have contributed to differences in IgG recognition profile.

The IDR8 recognition in gp41 was absent in UK003SG recipients due to the lack of gp41 in the MVA-C-gp120 vector. IDR8 recognition was still absent after the CN54gp140/GLA boost in UK003SG, but present in TMV01 after MVA-CMDR-gp150 and further boosted by CN54gp140/GLA. While we consider it unlikely, it is still possible that the inclusion of the gp41 IDR region present in the MVA-CMDR-gp150 encoded immunogen “deviated” IgG responses away from gp120 regions. Future studies need to address the hypothesis of possible “deviation” for vaccine induced IgG responses.

A high proportion of TMV01 recipients upon the MVA-CMDR-gp150 boosts recognized the identical V2 epitope that was correlated with protection from HIV-acquisition in the Rv144 trial (4, 5). There was no evidence that CN54gp140/GLA could boost this response in TMV01 recipients. The great V2 epitope sequence diversity between the MVA-CMDR-gp150 and CN54gp140 immunogen sequences may have contributed to this lack of boosting effect on an existing V2-epitope-specific IgG response in TMV01 vaccine recipients. Recognition of this epitope was however also completely absent in UK003SG recipients immunized exclusively with CN54 based immunogens in DNA, MVA and the CN54gp140 protein boost. Together these observations support the hypothesis that both, structural and sequence determinants inherent to the CMDR immunogen, but not present in the CN54 Env immunogens, contributed to the recognition of this V2 epitope in MVA-CMDR recipients. Interestingly, we also were unable to detect such V2-epitope specific IgG responses in most Tanzanian HIV seroconverters within 2 years of infection with subtype A and C molecular forms of the virus, suggesting that this response is fairly uncommon during early natural infections (unpublished data). Rv144 and Vax003 share the induction of this V2 epitope recognition with TMV01 recipients. However, only 10 of 91 tested V2 peptide variants were recognized at moderate strength in TMV01, which appears less cross-reactive as what was reported from Rv144 and Vax003 (4). In TMV01 recipients, recognized V2 peptide variants were identical or close to the CMDR immunogen sequence and—similar to what was reported for Vax003 and RV144—had a K in the position 169, which demonstrated a sieve effect in Rv144 (6). It is noteworthy that V2 epitope sequences are identical between MVA-CMDR, the Rv144 immunogens ALVAC-HIV and the recombinant AIDSVAX E (34). Hence, differences in V2 cross-recognition between our study and the study by Gottardo et al. (4) may be either linked to the boosting effect of the AIDSVAX recombinant protein boosts and/or to differences in the analysis pipeline applied. In addition, assessing vaccine-induced V2 recognition using V1V2-scaffold antigens of different clades appears to be more sensitive as compared to the linear peptide array approach taken here (5) and the comparatively poor cross-recognition reported here may therefore be underestimate true cross-recognition of the V2 region in TMV01. In summary, V2 recognition in TMV01, Vax003 and Rv144 was most likely induced by an identical immunogen sequences in V2. Both, gp120- and gp150-membrane anchored molecular, as well as both MVA-encoded (TMV01) and recombinant gp120 forms (AIDSVAX AE in Vax003) can therefore induce this V2 response. Hence, recognition of this V2 region in TMV01 recipients is probably more related to immunogen sequence and V2 structural information embedded in the primary aa sequence, and less related to the presence of the near native structure of Env encoded by MVA-CMDR (18).

The CN54gp140 protein boosted recognition of the V3 hypervariable region and further improved variant cross-recognition particularly in TMV01. It also had a measurable boosting effect in UKHVC03SG, where V3-specific recognition was already high after the MVA-C-gp120 boosts. The sequence homologous prime-boost regimen UK003SG induced better recognition of diverse variants of the V3 region as compared to the sequence heterologous TMV01 prime-boost regimen. This suggests that priming with heterologous Env immunogens does not necessarily improve variant recognition in the V3 region and rather argues against priming with a hypervariable immunogen in order to increase the depth of antigen variant recognition. While speculative, it might therefore be possible to improve IgG recognition of the V2 epitope variants by using more similar prime-boost vaccination regimens for V2.

In conclusion, the pattern of IgG recognition of individual linear antigenic regions in Env observed in these two trials overlapped with those in other trials and mostly targeted non-glycosylated regions of the inter-gp120 interface. Our results argue in favor of the hypothesis that structural differences between the MVA encoded Env immunogens—secreted gp120 vs. “native-like,” functional, cell membrane anchored gp150—as well as immunogen sequence differences during the prime-boost vaccination (monovalent vs. multivalent) contributed to this pattern. There was no evidence that prime-boosting with highly variable immunogens improved cross-recognition of linear antigenic variants in this study.

## Ethics Statement

The study documents for both trials were reviewed and approved by the relevant Ethical review boards [TaMoVac01: Institutional review boards of the Muhimbili University of Health and Allied Sciences, the Mbeya Medical Research Ethics Committee, the Tanzanian National Health Research Ethics Committee; UKHVC_Spoke03: NRES London—West London and GTAC Ethics Committee (13/LO/0115)]. The retrospective epitope mapping of IgG antibody responses within both trials was further approved by the Ethics comitee of the Ludwig-Maximilians-University of Munich. Participants of both trials gave fully informed written consent according to the Declaration of Helsinki before any study procedures were conducted. The TaMoVac01 trial is registered at the World Health Organization International Clinical Trials Registry with registration number PACTR2010050002122368. The UKHVC Spoke 03 trial was registered with the European Union Drug Regulating Authorities for Clinical Trials (EUDRACT TC 2012-003277-26) and Clinical Trials.gov (NCT01922284) and with the UK Clinical Trials Research Network (UKRN-14173).

## Author Contributions

YN, KH, and MA performed laboratory work, contributed to data analyses, and manuscript writing. SJ, AK, and ES contributed to clinical trials study coordination and manuscript writing. MM, AB, AJ, SM, AC, RT, RS, MR, MH, LM, MB, and JW contributed to the clinical trials studies. VH and UR contributed to designing R scripts for peptide array data analyses. JZ contributed to design and manufacturing of peptide microarrays. DP and RW contributed to the analyses and interpretation of peptide array data in the context of immunogen structure and to manuscript writing. GP contributed to peptide array design, phylogenetic sequence and data analyses and to manuscript writing. CG conceived the study, contributed to data analyses and interpretation and to manuscript writing.

### Conflict of Interest Statement

UR and JZ were employed by the company JPT Peptide Technologies. The remaining authors declare that the research was conducted in the absence of any commercial or financial relationships that could be construed as a potential conflict of interest.
