# Supplementary material for: Envelope-Specific Recognition Patterns of HIV Vaccine-Induced IgG Antibodies Are Linked to Immunogen Structure and Sequence
Source: Front Immunol. 2019 Apr 24;10:717. doi: 10.3389/fimmu.2019.00717 (PMC6492543; doi:10.3389/fimmu.2019.00717)
Supplement: Supplementary file 1 [file Table_1.pdf]

Table S1: Isolates utilised for the peptide array design

Patient Code (id)	Accession	Name	Subtype	Country	Sampling Year	Fiebig stage	Patient sex	Patient Health	Risk Factor	Coreceptor	Viral Load	Days from Seroconversion
R163(38637)	GU481385	06_RU_SP_R163_IorII_13	A6	RU	2006	1 or 2	F	acute infection	IV Drug User		316228	pre-seroconversion
Q842(1121)	AF407160	Q842-d12_PNS70d	A1	KE	1994		F	acute infection	Sex worker	CCR5	271220	28
374(9528)	AF041133	PIH374	B	FR	1995		M	acute infection	Homosexual			0
BORI0637(14179)	EU576282	BORId9_2F8	B	US	1990	2	M	acute infection	Male Sex with Male	CCR5	2400000	0
25925(13781)	EF117273	HIV_25925_2	C	IN	1999	3	M	acute infection	Heterosexual	CCR5	616531	45
0393(30635)	FJ444215	CHV0011210_0393-C3	C	MW	2003	4	F	acute infection	Heterosexual		12048485	early
426(3691)	AY231158	PHI426	01_AE	FR	1996		M	acute infection	Heterosexual		16767	30
127(3688)	AY231152	PHI127	02_AG	FR	1992		F	acute infection				30
