# Supplementary material for: Envelope-Specific Recognition Patterns of HIV Vaccine-Induced IgG Antibodies Are Linked to Immunogen Structure and Sequence
Source: Front Immunol. 2019 Apr 24;10:717. doi: 10.3389/fimmu.2019.00717 (PMC6492543; doi:10.3389/fimmu.2019.00717)
Supplement: Supplementary file 2 [file Table_2.pdf]

	MFI over all vaccinees			
	UK003SG		TMV01	
V3 Variant Sequence	post 2ndMVA	post CN54gp140	post 2ndMVA	post CN54gp140
RKGIHLGPGQTFYAT	24249	46742	5639	26781
RKGIHMGPGRAFYAT	834	2823	1155	8111
RKRIRIQRGPGRAFV	268	627	76	789
RKSIHIAPGRAFYAT	3949	11177	937	6106
RKSIHIGPGKAFYAA	34174	48823	1785	22568
RKSIHIGPGQAFYAT	39454	51943	6229	32784
RKSIHIGPGQAFYTT	42701	52636	4997	29122
RKSIHIGPGRAFYAT	120	168	216	293
RKSIHIGPGRAFYTT	20559	41641	1599	16351
RKSIHMGPGKAFYAT	1620	6872	1548	10114
RKSINIGPGRAFYAT	46028	52331	2661	34311
RKSINIGPGRAFYTT	40755	48250	3584	34981
RKSINIGPGRALYTT	45270	48566	529	27456
RKSIPIGPGRAFYAT	3973	18038	7999	28583
RKSIPIGPGRAFYTT	3204	14766	8108	27843
RKSIRIGPGQAFFAT	48692	54605	2695	35319
RKSIRIGPGQAFYAT	48698	54162	4352	36563
RKSIRIGPGQAFYTT	39648	53203	1524	22505
RKSIRIGPGQSFYAT	51757	53128	5265	49289
RKSIRIGPGQTFYAT	50904	52656	6470	53469
RKSIRIGPGQVFYAT	37535	49290	798	12490
RKSIRIGPGSTFYAT	47800	54793	359	20930
RKSISIGPGRAFYAT	41215	53121	3609	36920
RKSMRIGPGQTFYAT	8248	28908	5491	28466
RKSVHIGPGQAFYAT	723	6551	574	10077
RKSVRIGPGQAFYAM	41914	50533	7990	35545
RKSVRIGPGQAFYAT	32752	50480	4489	27422
RKSVRIGPGQTFYAT	50539	54490	4638	39986
RQSIRIGPGQTFYAT	95	1241	129	272
RQSTHIGPGQALYTT	55	345	62	901
RRSIHIGPGRAFYAT	2058	7572	883	6930
RRSINIGPGRAFYAT	10697	31353	2051	17467
RRSIRFGPGQAFYAT	4997	21575	2210	8746
RRSIRIGPGQTFYAT	48769	53735	2591	35544
RRSVRIGPGQTFYAT	27956	51420	2618	24331
RTSMRIGPGQVFYRT	2212	10509	2950	12870

Supplementary Table 2. Numeric data of Mean Fluorescence Intensity (MFI) values for the IgG recognition of HIV Envelope variable region 3 peptide variants as shown in the phylogenetic heat map of Figure 7.
