# Supplementary figures and images for: Envelope-Specific Recognition Patterns of HIV Vaccine-Induced IgG Antibodies Are Linked to Immunogen Structure and Sequence
Source: Front Immunol. 2019 Apr 24;10:717. doi: 10.3389/fimmu.2019.00717 (PMC6492543; doi:10.3389/fimmu.2019.00717)

Phylogenetic Heatmap of V2 peptide variant recognition in TMV01 recipients after MVA#2

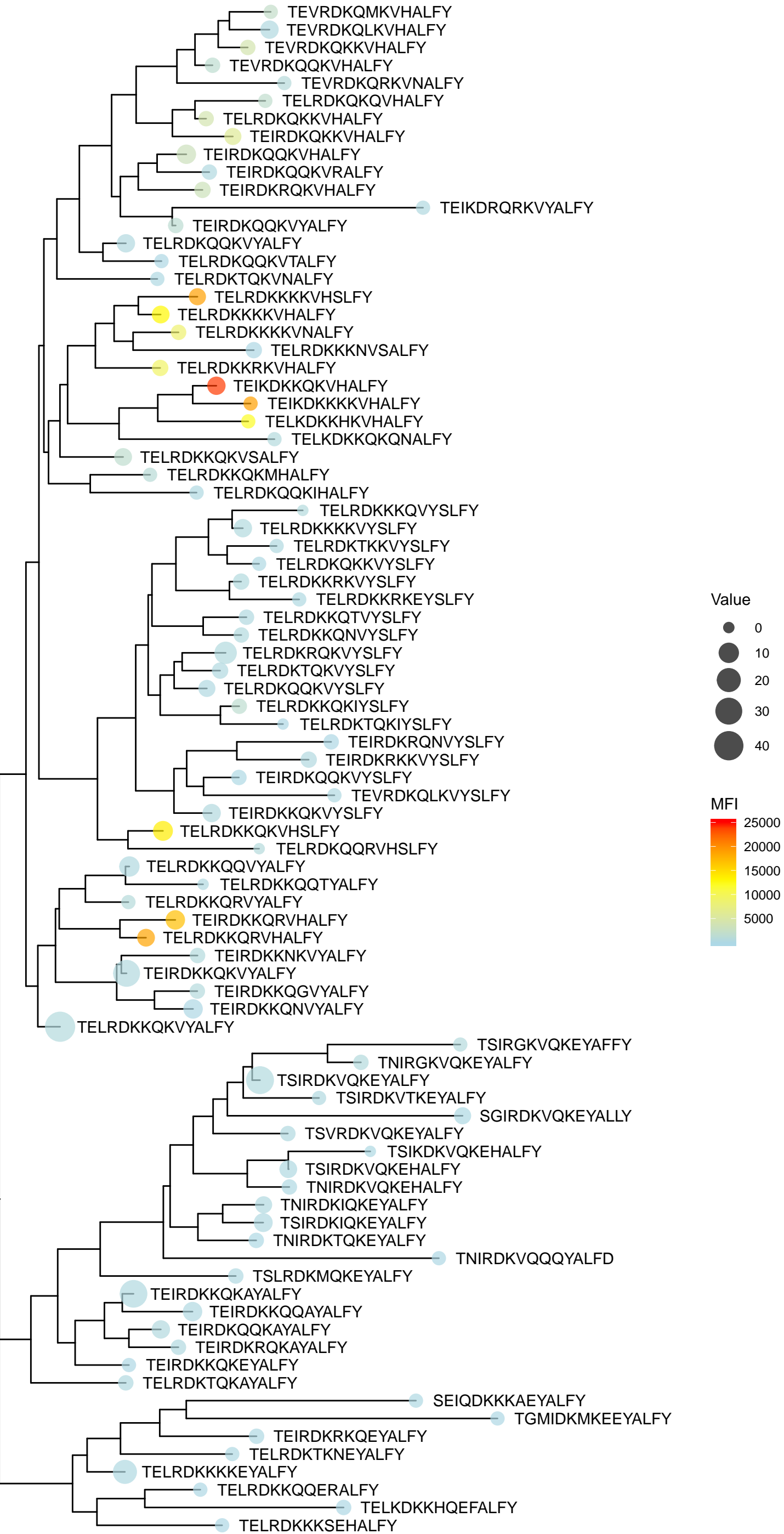

Supplement: Supplementary file 3 [file Data_Sheet_1.pdf]
